# Biofilm-Mediated Enhanced Crude Oil Degradation by Newly Isolated *Pseudomonas* Species

**DOI:** 10.5402/2013/250749

**Published:** 2013-03-05

**Authors:** Debdeep Dasgupta, Ritabrata Ghosh, Tapas K. Sengupta

**Affiliations:** Department of Biological Sciences, Indian Institute of Science Education & Research-Kolkata, Mohanpur Campus, Nadia 741252, India

## Abstract

The bioavailability of organic contaminants to the degrading bacteria is a major limitation to efficient bioremediation of sites contaminated with hydrophobic pollutants. Such limitation of bioavailability can be overcome by steady-state biofilm-based reactor. The aim of this study was to examine the effect of such multicellular aggregation by naturally existing oil-degrading bacteria on crude oil degradation. Microorganisms, capable of utilizing crude oil as sole carbon source, were isolated from river, estuary and sea-water samples. Biochemical and 16S rDNA analysis of the best degraders of the three sources was found to belong to the *Pseudomonas* species. Interestingly, one of the isolates was found to be close to *Pseudomonas otitidis* family which is not reported yet as a degrader of crude oil. Biodegradation of crude oil was estimated by gas chromatography, and biofilm formation near oil-water interface was quantified by confocal laser scanning microscopy. Biofilm supported batches of the isolated *Pseudomonas* species were able to degrade crude oil much readily and extensively than the planktonic counterparts. Volumetric and topographic analysis revealed that biofilms formed in presence of crude oil accumulate higher biomass with greater thickness compared to the biofilms produced in presence of glucose as sole carbon source.

## 1. Introduction

World marine ecosystem has been studied extensively since the second half of the last century. Oil spillage and oil pollution in marine environment have been a major threat to the ecosystem including the ocean life as well as to the human being through the transfer of toxic organic materials including polycyclic aromatic hydrocarbons (PAHs) into the food chain [1–3]. Presence of polycyclic aromatic hydrocarbons (PAHs) in soil and water is major problem as environmental contaminants and most of these PAHs are recalcitrant in nature. PAHs mean a potential risk to the marine animals as well as to the human health as many of them are carcinogenic [4]. Physical and chemical methods like volatilization, photooxidation, chemical oxidation, and bioaccumulation [5] are rarely successful in rapid removal and in cleaning up PAHs [6], and also these methods are not safe and cost effective when compared to microbial bioremediation. Bacteria have long been considered as one of the predominant hydrocarbon degrading agents found in the environment, which are free living and ubiquitous. Over twenty genera of bacteria of marine origin have been documented to be hydrocarbon degrading [7–9]. Bacteria belonging to subphyla *α*-, *β*-, and *δ*-*proteobacteria* [10–13] are well established to be of such nature. 

 One of the major factors that impedes the process bioremediation is bioavailability of hydrophobic contaminants to the hydrocarbon utilising microorganisms. Although numerous studies focused on biofilm reactor in the field of bioremediation [14], pollution of marine bodies by oil and other hydrocarbons solely during the oil spillage needs further attention in the context of bioavailability of microorganisms. It has been investigated earlier that this major limitation can be improved by exploiting chemotactic bacteria [15–17]. Microbial chemotaxis plays important role in surface colonization and biofilm formation [18, 19]. Microbes have a natural tendency to form multi-cellular aggregates being glued to form biofilm [20, 21]. Biofilm can be formed by single bacterial species or even by a group of bacteria, fungi, algae, and protozoa. The potential of microbial aggregates in the biofilm communities for bioremediation is always a safer and more adept method than planktonic microorganism as the biofilm matrix protects them during stress, and therefore organism gets a better chance of adaptation [22]. Interestingly, Klein et al. [23] reported that hexadecane assimilation by *Marinobacter hydrocarbonoclasticus* SP17 occurs through the formation of a biofilm at the alkane-water interface and how the cell behavior changes with the presence of utilizable or nonutilizable alkanes at the interface. The biofilm-based reactors furnish high microbial biomass accessible for better microbial activity than planktonic cells for other biological activities like biomineralization [24]. Recently the efficiency of biofilm-associated cells in degradation of naphthalene over planktonic had been elucidated for strain *Pseudomonas stutzeri* T102, and the survival of cells in petroleum contaminated soil is well documented [25]. Chandran and Das [26] demonstrated 97% degradation of diesel oil over a period of 10 days by yeast biofilm on gravel particle. Faster and intense depletion of linear and brunched hydrocarbon was observed in biofilm microbial community of *Alcanivorax borkumensis* [27]. Microbial consortia on gravel particle were found as conducive tools for self-cleaning of oily gulf coast throughout all the sites and season [28]. Biofilm community is diverse and relatively stable for longer period of time [29]. The film consortia were isolated from petroleum contaminated urban subway drainage system where they were capable of degradation at fifteen-degree centigrade [30]. The phenomenon of chemotaxis by the organisms towards the pollutants and the simultaneous attachment-detachment process maintains a constant load of biomass to the affected site in the water bodies. 

 Oil spillage has taken place in India for more than one instance. Notably, in early 2006, a Japanese tanker collided with a small Indian vessel 470 km west of the Nicobar and Andaman Archipelago, spilling over 4,500 tons of oil into the Indian Ocean. More recently, a ship carrying iron ore is reported to be spilling oil in the sea near Paradip Port (Orissa, India) since it has gone down under sea in September 2009. Kolkata Port and nearby areas like Haldia port, part of Bay of Bengal close to the ports and Haldia Refinery are major concerns for possible oil spillage due to everyday transport of fuel oil and other means since it is a major shipping corridor for eastern region of India. In spite of possible threat of contamination of water sources by spilled oil in these areas, little work has been done so far on presence and characterization of oil-degrading microorganisms, naturally existing in water sources near Kolkata port and nearby areas.

Our present work was to emphasize the multicellular aggregation of biofilm formation by naturally occurring hydrocarbon degrading strains from this region and to investigate the applicability of biofilm amendment on enhancement of biodegradation of crude oil. Briefly, microorganisms, capable to utilize crude oil as sole carbon source, were isolated from the water samples of the previously mentioned sources through serial enrichment culture technique. Based on better crude oil utilization ability, three of the isolated strains from the three mentioned sources were screened. The organisms were characterized and identified by biochemical test and 16S rDNA sequencing and further tested for utilization of various fuel oils and their ability to form biofilm. The volumetric and topological properties of biofilm near oil water interface were estimated by confocal laser scanning microscopy (CLSM). Gas chromatography-mass spectroscopic analysis was carried out to measure the effect of biofilm amendment on crude oil degradation in comparison to planktonic culture alone. 

## 2. Materials and Methods

### 2.1. Source of Microorganisms

Water samples were collected from Kolkata port of Hooghly River (22°32′N, 88.24′E), River Haldi at Haldia port (22°05′N, 88°03′E) and Bay of Bengal at Digha (21°37′N 87°25′E) for isolation of crude oil degrading microorganism. Water sample from Gomukh glacier (30°58′N, 78°55′E), the source of Ganges river at the altitude of 3819 m, was used as control nonpolluted water and tested for a presence of crude oil degrading bacteria.

### 2.2. Culture Enrichment Isolation and Characterization of Strain

The enrichment of crude oil degrading bacteria was carried out under aerobic condition with crude oil as sole source of carbon. Crude oil was obtained from Indian Oil Corporation Limited (IOCL, Haldia, West Bengal). The mineral salt media (MSM) [31] were amended with 1% crude oil (v/v), and enrichment of culture was carried out in three consecutive batches each having a span of 15 days and enriched by using previous growth as inoculums for the next. Bacterial growth was measured by using spectrophotometer (Chemito Instruments UV 2600) at 600 nm and compared with control without inoculation. Selective solid inorganic media (SSIM) [32] were inoculated by spreading 100 *μ*L of broth from last batch of enriched culture incubated at 30°C for 10 days. Representative pure colonies were isolated and further confirmed for oil degradation by growing in MSM media provided with 1% crude oil (filter sterilized using 0.2 *μ*m syringe filter). 

Selection of microorganisms was based on better ability to grow in presence of crude oil as sole source of carbon in growth media. The isolated microorganisms were tested for Gram staining and biochemical properties as described previously [33–35]. Motility tests were done by stabbing cells in semisolid nutrient agar (0.7% agar) [33]. 

### 2.3. Growth Characteristics in Different Oil and Biodegradation Analysis

Studies on growth characteristics of the isolated microorganisms were carried in Bushnell-Hass (Difco) media using crude oil, diesel, kerosene, unused engine oil (Bharat petroleum), and used engine oil (obtained from local service station). These oil samples were filter-sterilized using 0.2 *μ*m syringe filter and added into 50 mL of BH media (composition (gm/lit) MgSO_4_ (0.2), CaCl_2_ (0.02), KH_2_PO_4_ (1.0), (NH_4_)_2_HPO_4_ (1.0), KNO_3_ (1.0), FeCl_3_ (0.05) and 1% of oil sample), pH 7. The BH media were inoculated with isolated bacteria and incubated at 30°C under static condition for 5 days. Aliquots of bacterial cultures were collected, serially diluted, and plated on nutrient agar plates. The numbers of colonies were counted to determine bacterial growth in terms of colony forming units (CFU/mL).

For biodegradation studies gas chromatography-mass spectroscopic (GC-MS) analysis of crude oil was carried out. Isolated microorganisms were grown in 50 mL of BH medium (pH 7 ± .02) at 30°C for 15 days in presence of 1% crude oil as sole carbon source. After 15 days of growth, the residual crude oil components were extracted with equal volume of organic solvent dichloromethane (DCM). The aqueous phase and the organic phase were separated in separating funnel. The residual water from the organic phase was absorbed by anhydrous sodium sulphate (1 gm/10 mL). 10 microliter samples of the DCM extracts were then analyzed by GC-MS (Agilent 6890N GC-MS-5973N) with a column of 30 (m) × 0.25 (*μ*m) at a flow rate of 1.00 mL/min [36]. The samples were held at 60°C for 2 minutes initially and increased at the rate of 20°C/min to reach the final temperature of 325°C. The final temperature was held for 5 minutes. 50 mL of BH medium with 1% crude oil was also kept at 30°C for 15 days as control and crude oil components were extracted with DCM and analyzed by GC-MS as stated before. 

### 2.4. 16S rDNA Sequencing and Phylogenetic Analysis

A colony of each isolate was grown overnight in LB medium incubated at 30°C. 1 mL sample of each culture was centrifuged at 2300 g for 10** **min. The bacterial DNA was isolated using bacterial genomic DNA isolation kit (Chromous Biotech), and 16S rDNAs were amplified by using PCR master mix (Fermentas). Bacterial universal primers Forward-27f (5′-AGAGTTTGATCATGGCTCAG-3′) and Reverse-1492r (5′ TACGGYTACCTTGTTACGACTT-3′) were used for amplification [37]. The PCR products were purified with QIAquick Gel extraction kit (Qiagen). Nucleotide sequences were determined from the purified product by automated sequencer with an ABI PRISM II Dye Terminator Cycle Sequencing kit (Chromous-Biotech) with the same primers.

The identity of 16S rDNA sequences of isolates was determined by using the BLAST database search [38]. Eighteen sequences (first 6 hits for each isolate) of the cultivable organisms were procured from NCBI-Blast search, and the alignment was done by using CLUSTALX 1.82 software. The alignment was thoroughly checked in software SEAVIEW for any gaps and edited accordingly. A phylogenetic tree was constructed by neighbour joining method (Kimura 2-parameter) using MEGA v-4.0, and the tree was subsequently bootstrapped (random speed 64328, 1000 replicates).

### 2.5. Oil Biofilm Development and Quantification

Three isolated strains were first tested for biofilm formation on 18 mm glass cover slips being immersed in 15 mL BH media with 1% crude oil in 50 mL sterile falcon tubes. The organism was inoculated and incubated at 30°C for 4 days. The cover slips were recovered from the culture tubes, washed thoroughly in 1% saline solution aseptically, air-dried and Gram-stained. Formation of biofilm was viewed under 100X oil immersion objective using Nikon's DN100 microscope. The formation of biofilm on thin glass cover slips was also studied for hourly development of film by the strain KPW.1-S1 and stained at 6 different time intervals of growth at 6th, 12th, 18th, 24th, 36th and 48th hours. 

Biofilm load in presence of crude oil was estimated by the confocal laser scanning microscopic (CLSM) image stacks [39]. Briefly, the isolated strains were grown on glass surface in presence of 1% crude oil (glass slide: 25 × 75 mm) and 2% glucose (v/v) (cover glass: 12 × 12 mm), respectively, in BH media. The surface of the substratum was washed with PBS (1X) thrice and stained with 0.005% acridine orange (w/v) for 5 minutes in dark and washed twice. The slides were observed under Carl Zeiss CLSM-710 (Axio observer microscope version Z.1) using 488 nm excitation argon laser with MBS (main beam splitter) and emission wavelength detected from 493 to 586 nm. The image acquisition was done under X100 oil immersion lens (NA: 1.4). Series of measurement was taken at random vertically across the oil water interface (in case of crude oil) and air water interface in case of glucose. All samples were viewed from the clean side of the cover slip under oil, and the height was measured from these transects vertically from the base of cover slip to the top of the biofilm (frame size 512 × 512, 8-bit image, *Z* stacks interval 0.37*μ*m). Volumetric and topological parameters (thickness, biovolume, biomass, surface area, skewness, and kurtosis) of biofilm were calculated using the software provided (Zen 2010) along with the microscope.

### 2.6. Effect of Biofilm Amendment on Biodegradation

Oil degradation was also compared for biofilm amended and unamended planktonic cultures (with equal starter inoculums) with KPW.1-S1 and strain with biofilm defect DSW.1-S4. Glass slide of dimension 25 mm × 75 mm was immersed in 50 mL falcon tube containing 20 mL BH media with 1% crude oil and incubated for 15 days at 30°C. For biodegradation analysis other sets of batch cultures with and without biofilm carrying the residual crude oil were extracted with equal volume of organic solvent dichloromethane. The aqueous phase and oil were separated. The residual water was absorbed by anhydrous sodium sulphate (1 gm/10 mL). The extract was analyzed by gas chromatography-mass Spectroscopy (Agilent 6890N GC-MS-5973N) as described in the previous section. Percentage of degradation for ten detectable peaks (with respect to control) was calculated by the method described earlier [40].

### 2.7. Statistical Analysis

Graph plotting and multiple comparisons of optical densities were assessed by Origin 7 followed by SigmaPlot software version 11, San Jose, California, USA. The data were expressed as mean ± standard error.

## 3. Results

### 3.1. Enrichment and Screening of Organism

**Figure 1:**
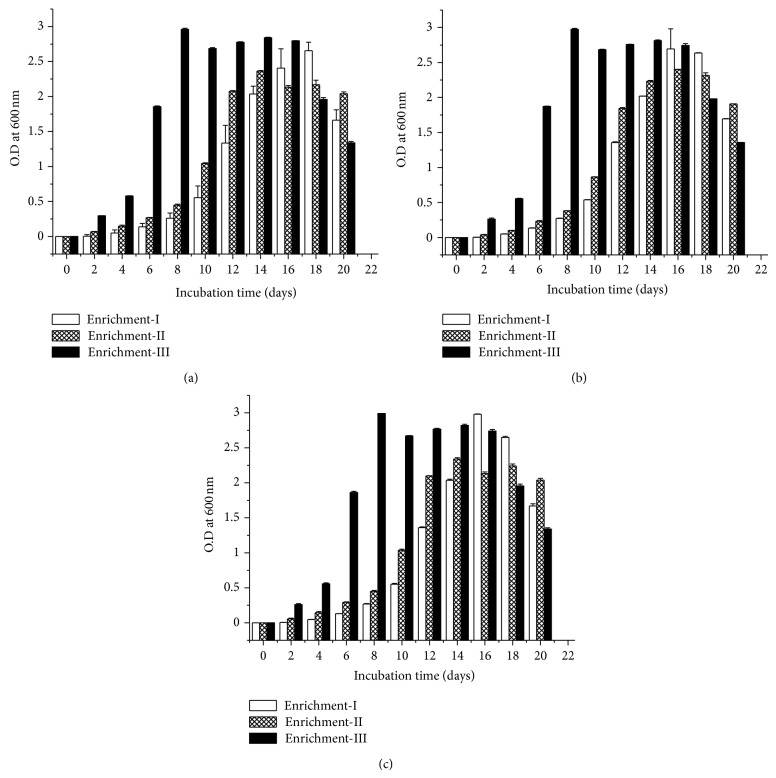
Enrichment of crude oil-degrading bacteria. Bacterial populations in water samples, collected from (a) Kolkata Port, (b) Haldi River and (c) Digha site at Bay of Bengal were subjected to three-step enrichment process in MSM in presence of crude oil as sole carbon source. In all the cases the standard error values ranged from 0.1% to 1%.

During incubation of water samples (collected from Kolkata Port, Haldia Port, and Bay of Bengal areas) in MSM media (containing crude oil as sole carbon source) no visual change in turbidity due to bacterial growth was observed till the third day of incubation. The optical density (for bacterial growth) kept increasing slowly for the next 16–18 days. The second enrichment using inoculums from the first enrichment showed a little early response with a shorter lag period of growth, and the culture reached stationary phase of growth within 14–16 days. Finally, in the third serial enrichment, culture inoculated from the second batch showed an early response, and significant growth was observed in the second day onwards and reached stationary phase of growth within 7 days (Figures 1(a)–1(c)). Interestingly, water sample, collected from Gomukh glacier, showed no growth even after 6 weeks of incubation in MSM medium, containing crude oil as sole carbon source (data not shown).

From the third batch of enriched cultures, pure colonies were isolated on SSIM crude oil agar plates. Total of 36, 30, and 16 pure colonies were thus obtained from three water samples collected from Kolkata port, Haldi River water, and Bay of Bengal near Digha site, respectively. These colonies (in duplicate) were restreaked on nutrient agar (NA), and they were differentiated by colony characteristics based on morphology and pigmentation. The distinct 13 colonies with unique characteristics were isolated as pure culture in NA media. All isolated colonies were tested for further confirmation of their ability of oil degradation, and three isolates from three different sources were selected based on their rapid growth in presence of crude oil as sole carbon source (data not shown). The organisms were named as KPW.1-S1 (isolated from Kolkata Port water, MTCC 10087), HRW.1-S3 (isolated from Haldi River water, MTCC 10088), and DSW.1-S4 (isolated from Digha sight of the shore of Bay of Bengal, MTCC 10089) and currently deposited at Microbial Type Culture Collection and Gene Bank (MTCC), India. 

### 3.2. Growth Characteristics in Different Oil and Biodegradation Analysis

**Figure 2:**
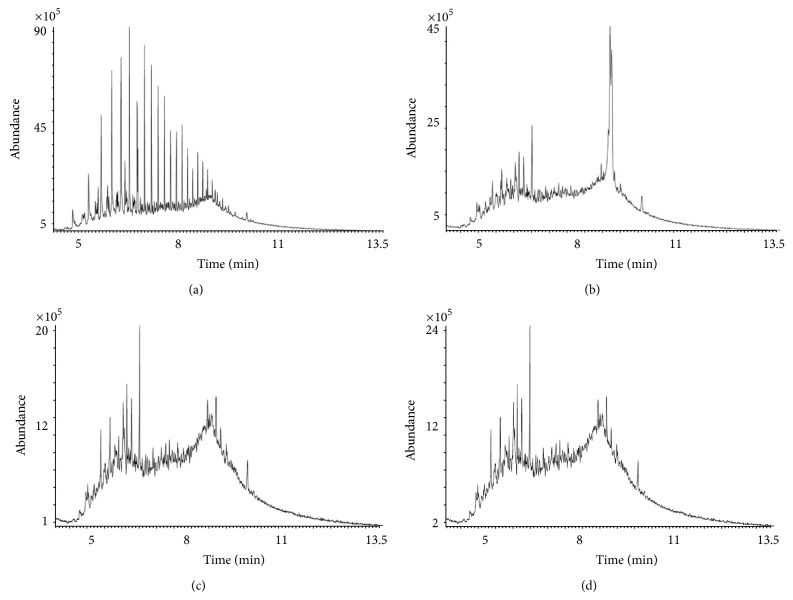
Biodegradation of crude oil (at 30°C, 15-day incubation) analyzed by GC-MS. (a) Without microorganism (control) and with organisms (b) KPW.1-S1, (c) HRW.1-S3, (d) and DSW.1-S4.

The selected bacteria (KPW.1-S1, HRW.1-S3, and DSW.1-S4) were subjected to grow in presence of various other oils as carbon source. The bacterial growth was found to be uneven depending on the bacterial species and oil type (Table 1). Significant difference was observed between the growth of the three strains in case of crude oil and hexadecane (*P* < 0.05). The strain KPW.1-S1 showed best growth in presence of hexadecane as carbon source and the DSW.1-S4 in presence of crude oil and higher hydrocarbons present in engine oil. The kerosene showed to be the least supportive as a carbon source, and it had no significant effect on difference of growth among the three strains (*P* > 0.05). The strain HPW.1-S3 showed average growth rate and crude oil degradation (Figure 2(c), Table 1). These results clearly indicate that different oils were degraded and utilized by all the strains in various proportions, depending on the complexity and aliphatic and aromatic nature of the sample oil dependent on bacterial species as well. 

Organisms KPW.1-S1, HRW.1-S3, and DSW.1-S4 were grown in Bushnell-Hass (BH) media in presence of crude oil as only carbon source at 30°C for 15 days. The hydrocarbon profile in the growth media after 15 days of growth was analyzed by GC-MS and compared with the hydrocarbon profile from a control flask where the BH medium and crude oil were kept together for 15 days under identical conditions. The hydrocarbon profile obtained by GC-MS analysis showed the relative abundance of various hydrocarbons in the complex mixture (Figure 2). The control sample (Figure 2(a)) shows the presence of various hydrocarbons in the unresolved complex mixture. All the isolated bacteria were able to reduce at least 50% of relative abundance of various hydrocarbons present in crude oil compared to control experiment (Figures 2(b)–2(d)) within 15 days of incubation. Interestingly, bacterial strains isolated from Kolkata port (KPW.1-S1) and Digha area of Bay of Bengal (DSW.1-S4) showed minimum and maximum overall hydrocarbon degradation abilities, respectively.

### 3.3. Taxonomic Identification of Bacterial Strains

**Figure 3:**
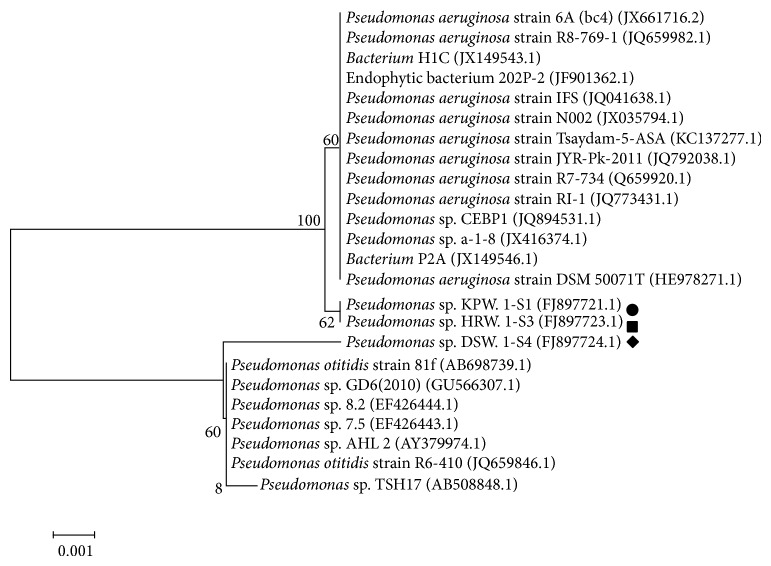
Phylogenic tree of all sequenced *β*-*Proteobacteria* (•) KPW.1-S1, (■) HRW.1-S3, (♦) DSW.1-S4. The bootstrap values are indicated for the major nodes.

Isolated three organisms (KPW.1-S1, HRW.1-S3, and DSW.1-S4) were tested for a series of biochemical and morphological tests (Table 2). All three organisms, KPW.1-S1, HRW.1-S3, and DSW.1-S4, were found to be Gram-negative, citrate positive, rod-shaped (similar to coccobacillus shape), and motile. When crude oil was used as carbon source, all the isolated organisms showed similar growth characteristic at different temperatures ranging from 20°C to 37°C with an optimum temperature at 30°C. Further analysis of 16S rDNA gene sequences was done for taxonomic identification. Amplification and sequencing of 16S rDNA followed by phylogenetic analysis (together with biochemical and morphological analyses as described in Table 2) revealed that the isolated strains (KPW.1-S1 (FJ897721), HPW.1-S3 (FJ897723), and DSW.1-S4 (FJ897724)) belong to phylum Proteobacteria, class Betaproteobacteria [41], and genus *Pseudomonas *(Figure 3). The strains KPW.1-S1 and HPW.1-S3 are the closest neighbour of *Pseudomonas aeruginosa* strain Tsaydam-5-ASA (KC137277.1), whereas DSW1-S4 is nearest to the candidate of* Pseudomonas otitidis *strain 81f (AB698739.1).

### 3.4. Oil Biofilm Development

**Figure 4:**
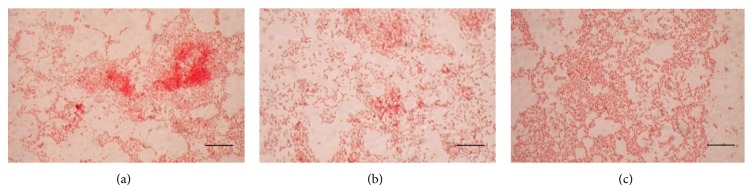
(a) Oil biofilm formation on glass surface by the three isolates (a) KPW.1-S1, (b) HRW.1-S3, and (c) DSW.1-S4. Bars, 23 *μ*m.

**Figure 5:**
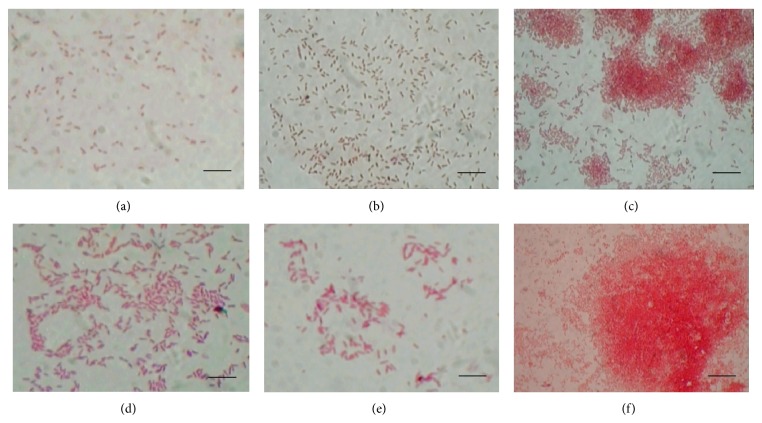
Hourly development of biofilm on thin glass cover slip at different time interval by KPW.1-S1 at (a) 6th, (b) 12th, (c) 18th, (d) 24th, (e) 36th, and (f) 48th hours. Bars, 23 *μ*m.

Preliminary studies demonstrated that the isolated bacterial strains possess the ability to form biofilm on glass surface when grown in BH medium in presence of crude oil as sole carbon source (Figures 4(a)–4(c)). Interestingly, a thick biomass was observed to be aggregated near the oil water interface on the glass bioreactor. Transitional episode of oil biofilm development was observed vividly using light microscopy. The initial event of bacterial attachment was found in the first sixth hour of growth (Figure 5(a)). In the next 6 hours, the cells start forming nascent cell cluster being cemented on the glass substratum (Figure 5(b)). At around 18 hours of growth cell clusters become more mature and initiate aggregation (Figure 5(c)). This was followed by evacuation and release of cells from the matrix with further incubation (Figures 5(d)–5(f)). Interestingly, KPW.1-S1 cells were again able to form well-defined biofilm on the same glass surface at around 48 hours of incubation (Figure 5(f)). 

### 3.5. Quantification of Biofilm by Confocal Laser Scanning Microscopy

**Figure 6:**
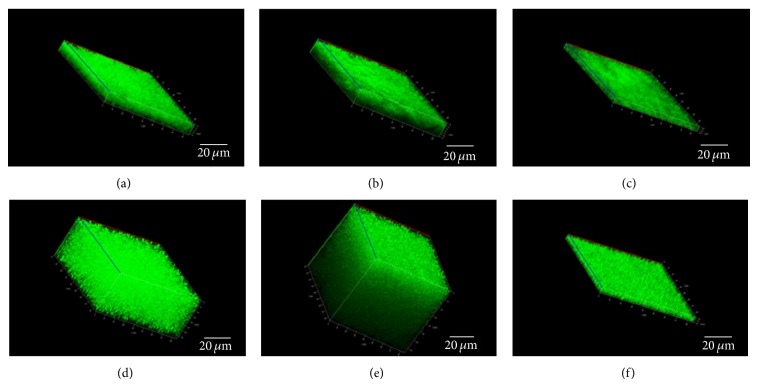
Confocal laser scanning microscopy image of biofilm hive near air water interface by three isolates (a) KPW.1-S1, (b) HRW.1-S3, and (c) DSW.1-S4 growing in presence of glucose as carbon source. Similar image was taken in 1% crude oil by the three isolates (d) KPW.1-S1, (e) HRW.1S3, and (f) DSW.1-S4 to compare the affinity of biofilm formation in presence of oil. Biofilms with maximum thicknesses achieved by the three isolates in presence of crude oil are represented in the figure. Bars, 20 *μ*m.

Volumetric and topologic quantification of biofilm formations by the isolated *Pseudomonas* species at oil-water interface was carried out by confocal laser scanning microscopy (CLSM). All of the three *Pseudomonas* strains tested showed ability to form biofilm near air-water interface when grown in presence of glucose as sole carbon source (Figures 6(a)–6(c)). The average thickness of KPW.1-S1 and HRW.1-S3 was found to be 10 and 12 *μ*m in presence of glucose whereas it was found to be 1.5 *μ*m in case of the third strain DSW.1-S4 (Table 4). Interestingly, enhanced biofilm production was observed when the *Pseudomonas* cells were grown in presence of crude oil as sole source of carbon (Figures 6(d)–6(f)). The average thicknesses of KPW.1-S1, HRW.1-S3, and DSW.1-S4 were found to be 22, 26, and 4.5 *μ*m, respectively, in presence of crude oil representing at least 2-fold increase of biofilm thickness for all the strains (Table 4). These results clearly showed that presence of crude oil in growth medium enhanced biofilm production by all of the three strains, although the DSW.1-S4 strain has lesser potential to form biofilm under the experimental conditions. Thus, the spatial biomass accumulated near the oil water interface by KPW.1-S1 and HRW.1-S3 was observed to be 14–18 units per unit area, and for the low biofilm former DSW.1-S4, spatial biomass accumulation was 1-2 units per unit area. In presence of crude oil, the maximum thickness of biofilm obtained by KPW.1-S1 and HRW.1-S3 was 35 and 73 *μ*m (Figures 6(d)–6(f)) whereas the strain possessing biofilm defect could develop up to 4.5 *μ*m of maximum thickness. 

To further substantiate our finding, topological parameters of biofilm load were investigated through the measurement of CLSM. Here, the mean thickness and biomass were found inversely proportional to skewness (*S*_ku_). Since higher skewness indicates lack of porosity [42], it is likely that lesser skewness and hence greater porosity enable biofilm to access higher nutrient and other essential factors which attributes in greater thickness of biofilm. The statement is validated in six cases comprising three strains and two conditions (Table 4). Strikingly, no significant difference was observed in other topological parameters, kurtosis (*S*_sk_) which implies that adherence of organism with substratum was more or less similar in all the six cases irrespective of strain with profound biofilm forming ability (KPW.1-S1 and HRW.1-S3) or strain DSW.1-S4 with lesser potential to make thick biofilm. 

### 3.6. Comparative Degradation Study

**Figure 7:**
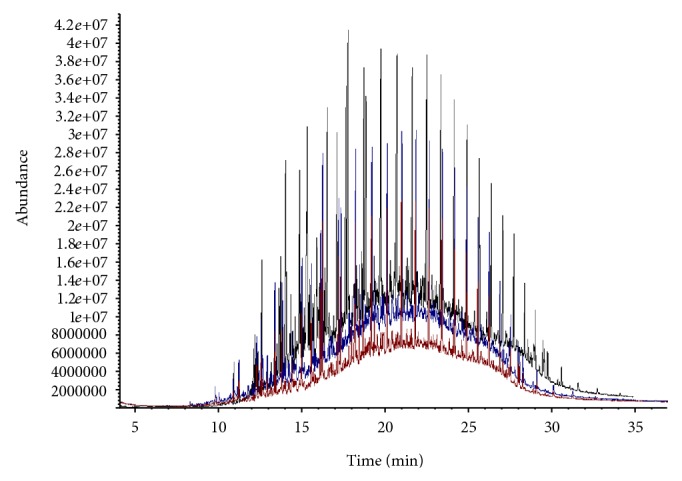
GC-MS analysis of oil degradation record of strain DSW.1-S4 for biofilm amended and unamended conditions. Chromatogram showing decrease in peak height and corresponding area. Control (black), Planktonic only (blue), and biofilm + planktonic cells (Red).

The aggregation of cells near oil-water interface observed through CLSM prompted to examine the relation between biofilm formation at the oil-water interface and utilization/degradation of crude oil components by the *Pseudomonas* isolates. A comparative account of GC-MS profiles of hydrocarbons present in crude oil was analyzed in presence and absence of substratum support. Biofilm forming strain KPW.1-S1 and biofilm defective DSW.1-S4 were taken into account for oil degradation analysis. The GC-MS result shows that the relative abundance of the peak reduced considerably for both of the strains depending on potential of oil degradation (Figure 7). Oil degradation in terms of reduction of total numbers of hydrocarbon peaks and depletion of total area of chromatogram of various components of crude oil was observed with the introduction of biofilm amendment. 20 ± 10% to 40 ± 10% increment of degradation was achieved in presence of matrix enclosed biofilm in comparison to planktonic cells alone for both of the strains. Short chain hydrocarbon peaks were out of detection limit. From the percentage of degradation measured using the method described earlier, it is evident that increased degradation of individual hydrocarbon is mediated or induced by the biofilm near oil air-water interface (Figure 7). Additionally for the strain KPW.1-S1 the biofilm assisted culture could target the short chain low molecular weight hydrocarbons (Table 3, Figure 7), whereas the strain DSW.1-S4 could successfully degrade both short and long chain hydrocarbons present in crude oil efficiently. 

## 4. Discussion

Ability to form biofilm on various surfaces is always advantageous for the microorganisms in terms of survival, metabolism, adaptation, and propagation [43, 44]. One of the major limitations faced in the process of bioremediation is the bioavailability of organic compounds on site [45]. Early studies that indicate biofilm forming bacteria can be employed to overcome this limitation although the application of steady-state biofilm in bioremediation is not well established. Studies indicate that biofilm-mediated bioremediation is a proficient approach and safer option since cells in biofilm have better chance of survival and adaptability especially during the stressed conditions [21, 46]. Establishment of biofilm on gravel particles and glass slides was reported previously [46] where the artificially glued microorganisms showed excellent attenuation of crude oil in liquid waste in batch culture. Vaysse et al. [44] showed altered profile of expressed proteins, specifically type VI secretion system in biofilm forming *Marinobacter hydrocarbonoclasticus SP17* at alkane-water interface. 

Crude oil degrading bacteria were unruffled from three different locations which are the prominent risk zones of oil contamination. Three-step enrichment process was employed to enrich and isolate microorganisms with greater degrees of oil-degrading capabilities. These bacteria were then preferred for additional characterization and identification. Out of the selected organisms, KPW.1-S1, HRW.1-S3, and DSW.1-S4 were isolated from water of Kolkata Port, Haldi River, and Digha at Bay of Bengal, respectively. All three bacteria were Gram-negative, motile, oxidase positive *coccobacilli* in nature, and were preliminarily identified as of class Betaproteobacteria.

Ability of the isolated bacteria for utilization of crude oil as carbon source was investigated by GC-MS analysis. The data revealed that the isolated strains could be able to reduce different hydrocarbons present in crude oil samples up to 50% within 15 days of growth under laboratory conditions (Figure 2). The isolated organisms also showed their ability to use other complex oils like petrol, diesel, and kerosene as carbon sources as well (Table 2). It is interesting to note that 16S rDNA sequence and phylogenetic analysis revealed that KPW.1-S1 and HRW.1-S3 are closest to *Pseudomonas aeruginosa* and DSW.1-S4 to *Pseudomonas otitidis* (Figure 3). Previous studies on naturally existing oil-degrading bacteria in these regions were elucidated although the molecular phylogenetic analysis based on 16S rDNA sequencing was not initiated [47]. Thus DSW.1-S4 is a novel *Pseudomonas* sp. in terms of its ability to degrade crude oil since there is no report so far for crude oil degradation ability of *Pseudomonas otitidis* or any closely related species of *Pseudomonas otitidis*, although, recently, Venketaswar Reddy et al. [48] reported a newly isolated *Pseudomonas otitidis* as a potential biocatalyst for polyhydroxyalkanoates (PHA) synthesis. Moreover, results of the present study strongly suggest the existence of hydrocarbon degrading consortia in the river water sample although the degradation of crude oil by the strains was limited to 50% based on the peak heights (Figure 2). It was presumptuous to assume that the major factor that governs the low rate of degradation was bioavailability of hydrocarbon to the microbial biomass. To address this limitation, we developed a laboratory scale steady-state biofilm reactor which circumvents the limitation to an appreciable extent as biofilm formation near oil-water interface accumulates substantially higher amount of biomass. 

The isolated *Pseudomonas *strains in this study showed their ability to form biofilm on glass surface in BH medium in presence of crude oil as the only carbon source (Figures 4(a)–4(c)). In addition to this, the chemotaxis elucidated by the bacteria adhering on biofilm could also support the fact of bacterial motility towards the crude oil and other pollutants present in it (data not shown). Moreover, there is a wealth of evidence supporting the fact that biofilm development and maturation follow a cycle of attachment and release. The time lapse image of oil biofilm development indicated that the multicellular aggregation near oil-water interface cycles at regular interval. These data further supports the fact that the biofilm amendment presents the hydrocarbon degrading consortia to the oil and toxic compounds floating on the aqueous surface for a prolonged duration. It has been documented earlier that the mass-transfer limitations that impede the bioremediation process can be overcome by cells displaying chemotaxis that can sense chemicals such as those adsorbed to soil particles in a particular niche and swim towards them [46]. Thus we tested the effect of oil biofilm (static) amendment on the overall degradation of crude oil by GC-MS analysis and characterized the biofilm formation at oil water interface by confocal laser scanning microscopy (CLSM).

First the biofilm forming ability of the three isolates was estimated by 96-well microtitre assay method in presence of various carbon source including glucose, glycerol, and hexadecane (data not shown). Biofilm load in presence of crude oil was not measured by this procedure as oil interfered with the assay process. Based on the characteristic pattern of biofilm development of the isolated strains, KPW.1-S1 and biofilm defective DSW.1-S4 were selected for oil degradation analysis. Our hypothesis got more impetus with GC-MS profile of the oil degradation in presence and absence of biofilm amendment. The calculation of percentage of degradation also suggests that the biofilm amended culture could successfully degrade the individual hydrocarbon with much greater efficiency (Table 3). Notably, the overall degradation was enhanced by 20%–40% for both of the strains when amended with matrix enclosed biofilm cells in comparison to planktonic cells alone (Figure 7). The comparative study of biofilm formation showed that the oil biofilm can accumulate a large number of hydrocarbon degrading organisms near the oil water interface. This biofilm hive in oil had unusually higher biomass compared to the film produced in glucose as carbon source. The degradation of individual hydrocarbons was also tested in representative ten peaks where the similar reduction of peak area was observed. Although the enhanced degradation by biofilm defective strain DSW.1-S4 was counterintuitive, the comparative GC-MS data suggests that the strain is better degrader when compared with others, and therefore the oil degradation was further enhanced by accumulated cells of DSW.1-S4 enclosed in biofilm near oil water interface. Additionally biofilm formation follows a dynamic cycle of attachment and release from its substratum during its meal on oil observed by Gram staining at different time points (Figures 5(a)–5(f)). The phenomenon is also reflected in its mean thickness and total biomass formation observed by confocal imaging of biofilm (Table 4). The three isolated strains varied in thier total thicknesses depending on the phase in which they were stained and photographed in independent experiments. The maximum thickness achieved by all the three strains was found higher than the mean due to the cycle of attachment and release. These findings prompted us to examine the overall degradation profile in a span of fifteen days by the biofilm amended and unamended conditions. The GS-MS data strongly supports the fact that the greater degradation is due to higher population density in the biofilm amended condition. 

The topological parameters of the biofilm architecture also highlight the intrinsic properties of these films. The mean thickness and average biomass calculated from confocal imaging software correlated with our biofilm assay result. The thickness and biomass were highest for KPW.1-S1 and HRW.1-S3 whereas they were least for DSW.1-S4. The test was repeated for biofilm image obtained using glucose as carbon source. From the result it was evident that the multicellular aggregation was significantly higher in presence of crude oil. The greater skewness value of the poor biofilm forming strain DSW.1-S4 makes the film more porous. The higher standard deviation relates the higher heterogeneity of the biofilm for the all the three strains. The kurtosis value for all the three strains was roughly the same making the adhesion potential nearly similar. 

 Hence, from the previous finding it was conclusive that in water bodies natural oil degrading strains capable of formation of biofilm degrade oil much faster and efficiently when compared to planktonic cells alone. Recently Al-Bader et al. [49] depict the role of phototrophic, diazotrophic, and hydrocarbon-utilizing bacterial biofilm consortia leaving the promise of bioremediation of aquatic hydrocarbon pollutants. Secondly, the enhancement of degradation is profound in all oil degrading naturally exiting strains with various degrees of biofilm forming capacity. These results tempt us to speculate that natural oil degrading strains can accumulate near oil water interface during oil spillage and efficiently circumvent the limitation of bioavailability of in situ bioremediation to a considerably greater extent. 

## 5. Conclusion

The results in the present study consolidate our finding that potent hydrocarbon degrading bacterial consortia exist naturally in the water body near Kolkata port, Haldia Refinery, and nearby areas in the eastern regions of India in contrary to the water sources from Himalayan Glacier where pollution through various sources is negligible. This signifies that the hydrocarbon degrading bacteria exist or evolved to exist with the ever increasing intensity of marine pollution. *Pseudomonas *strains, isolated from water sources near Kolkata port, Haldi River, and Digha Sea shore, showed that their ability to degrade various complex hydrocarbons and biofilms formed by the isolated bacteria could enhance degradation ability. Therefore, these isolated *Pseudomonas *strains could be considered for future use for bioremediation of contaminated spilled oil in water sources. However, further studies are needed to evaluate the potential of the isolated strains to degrade hydrocarbons in situ, in natural environmental conditions. Thus, the oil degradation capability, ability to form biofilm, greater survival in the nutrient stressed condition, cycle of attachment and release of biofilm-associated cells, and cooperative nature of these natural isolates could be exploited as a better option for bioremediation technology. This could be equally applicable for any *Pseudomonas* or other bacterial strains ubiquitously available in nature having the previously mentioned criteria, and the technology could be further developed for targeting of any pollutants present on earth creating enormous environmental and health hazards. 

## Figures and Tables

**Table 1:** Growth characteristics of isolated organisms by utilization of various oil/hydrocarbon as sole carbon source.

Oil/hydrocarbon	Colony forming unit/mL		
	Strain		
	KPW.1-S1	HRW.1-S3	DSW.1-S4
Crude oil	(1.5 ± 0.17) × 10^8^	(6.8 ± 0.11) × 10^8^	(1.4 ± 0.09) × 10^9^
Diesel	(6.7 ± 0.072) × 10^8^	(6.1 ± 0.08) × 10^8^	(6.5 ± 0.12) × 10^8^
Kerosene	(7.7 ± 0.06) × 10^7^	(1.6 ± 0.04) × 10^7^	(1.7 ± 0.07) × 10^8^
Hexadecane	(2.94 ± 0.13) × 10^9^	(4.4 ± 0.02) × 10^8^	(2.4 ± 0.07) × 10^8^
Engine oil	(6.1 ± 0.07) × 10^8^	(6.4 ± 0.11) × 10^8^	(3.6 ± 0.07) × 10^8^

**Table 2:** Preliminary biochemical tests for isolated bacterial strains.

Characteristics	KPW.1-S1	HRW.1-S3	DSW.1-S4
Gram nature	Negative	Negative	Negative
Shape	Rod	Coccobacillary	Coccobacillary
Diffusible pigments	+	+	−
Motility	+	+	+
Citrate utilization	*++ *	*++ *	*++ *
Lysine utilization	*+ *	+	+
Ornithine utilization	+	+	+
Urease test	*− *	*− *	*− *
Phenylalanine test	NC	NC	NC
Nitrate reduction	NC	NC	NC
H_2_S production	*+++ *	*++ *	*+ *
Production of acid by utilization of sugar∗			
(i) Glucose	*− *	*− *	*− *
(ii) Adonitol	*− *	*− *	*− *
(iii) Lactose	*− *	*− *	*− *
(iv) Arabinose	*− *	*− *	*− *
(v) Sorbitol	*− *	*− *	*− *

NC: not conclusive.

∗The test attributed the change of the color of pH indicator by production of acid and gas when grown in presence of various sugars.

**Table 3:** Percentage degradation of individual hydrocarbon present in crude oil.

Peak no.	Retention time (minute)	Percentage degradation			
		*P*/KPW.1-S1	*B* ^*L*^/KPW.1-S1	*P*/DSW.1-S4	*B* ^*L*^/DSW.1-S4
1	12.635	42.08	69.87	48.70	70.80
2	14.066	81.69	89.12	76.57	87.45
3	14.953	90.76	92.88	93.84	95.76
4	16.55	9.81	32.35	80.45	87.22
5	17.808	69.99	81.87	73.74	85.62
6	18.75	55.72	73.28	57.25	74.04
7	19.77	56.70	70.41	69.69	81.95
8	22.52	65.32	69.84	59.29	70.60
9	23.36	40.11	67.75	58.54	68.52
10	27.077	57.19	58.77	41.30	65.98
Overall∗	10.97–29.48	42.93	66.35	47.93	75.45

*P* represents degradation of hydrocarbon without amendment of biofilm (only planktonic).

*B* ^*L*^ represents degradation of hydrocarbon with amendment of biofilm (planktonic + biofilm).

∗Represents overall degradation of all the hydrocarbon considering the total area of control and samples under different conditions.

**Table 4:** Mean thickness, biomass, substratum coverage, kurtosis (*S*_ku_), and skewness (*S*_sk_) of biofilm of *Pseudomonas* sp. KPW.1-S1, HRW.1-S3, and DSW.1-S4.

Carbon source	Strain	Avg. thickness (*μ*m)	Total biomass (*μ*m^3^/*μ*m^2^)	Kurtosis (*S*_ku_)	Skewness (*S*_sk_)	Max. thickness (*μ*m)
	KPW.1-S1	22.36 ± 0.98	14.17 ± 1.71	3.5 ± 0.43	−0.68 ± 0.31	35
Crude oil	HWR.1-S3	26.51 ± 1.2	18.45 ± 1.36	4.19 ± 1.2	−1.01 ± 0.33	73
	DSW.1-S4	4.55 ± 1.6	1.6 ± 0.55	4.06 ± 0.2	0.86 ± 0.16	24
	KPW.1-S1	12.5 ± 0.89	9.33 ± 0.9	3.91 ± 0.5	−0.49 ± 0.34	23
Glucose	HWR.1-S3	10.32 ± 0.37	6.7 ± 0.49	2.72 ± 0.3	−0.46 ± 0.15	12.6
	DSW.1-S4	1.51 ± 0.08	0.9 ± 0.08	3.66 ± 0.2	0.48 ± 0.15	3.2

Values are means of data from 8 image stacks. The standard error is calculated as the square root of the mean of the variances of each of the four groups (image stacks from two glass slides in two independent experiment rounds).
